# Metabolic syndrome and risk of Parkinson disease: A nationwide cohort study

**DOI:** 10.1371/journal.pmed.1002640

**Published:** 2018-08-21

**Authors:** Ga Eun Nam, Seon Mee Kim, Kyungdo Han, Nan Hee Kim, Hye Soo Chung, Jin Wook Kim, Byoungduck Han, Sung Jung Cho, Ji Hee Yu, Yong Gyu Park, Kyung Mook Choi

**Affiliations:** 1 Department of Family Medicine, Sahmyook Medical Center, Seoul, Republic of Korea; 2 Department of Family Medicine, Korea University Guro Hospital, College of Medicine, Korea University, Seoul, Republic of Korea; 3 Department of Medical Statistics, College of Medicine, The Catholic University of Korea, Seoul, Republic of Korea; 4 Division of Endocrinology and Metabolism, Department of Internal Medicine, College of Medicine, Korea University, Seoul, Republic of Korea; Chinese University of Hong Kong, CHINA

## Abstract

**Background:**

The association of metabolic syndrome (MetS) with the development of Parkinson disease (PD) is currently unclear. We sought to determine whether MetS and its components are associated with the risk of incident PD using large-scale cohort data for the whole South Korean population.

**Methods and findings:**

Health checkup data of 17,163,560 individuals aged ≥40 years provided by the National Health Insurance Service (NHIS) of South Korea between January 1, 2009, and December 31, 2012, were included, and participants were followed up until December 31, 2015. The mean follow-up duration was 5.3 years. The hazard ratio (HR) and 95% confidence interval (CI) of PD were estimated using a Cox proportional hazards model adjusted for potential confounders. We identified 44,205 incident PD cases during follow-up. Individuals with MetS (*n =* 5,848,508) showed an increased risk of PD development compared with individuals without MetS (*n =* 11,315,052), even after adjusting for potential confounders including age, sex, smoking, alcohol consumption, physical activity, income, body mass index, estimated glomerular filtration rate, and history of stroke (model 3; HR, 95% CI: 1.24, 1.21–1.27). Each MetS component was positively associated with PD risk (HR, 95% CI: 1.13, 1.10–1.16 for abdominal obesity; 1.13, 1.10–1.15 for hypertriglyceridemia; 1.23, 1.20–1.25 for low high-density lipoprotein cholesterol; 1.05, 1.03–1.08 for high blood pressure; 1.21, 1.18–1.23 for hyperglycemia). PD incidence positively correlated with the number of MetS components (log-rank *p <* 0.001), and we observed a gradual increase in the HR for incident PD with increasing number of components (*p <* 0.001). A significant interaction between age and MetS on the risk of incident PD was observed (*p* for interaction < 0.001), and people aged ≥65 years old with MetS showed the highest HR of incident PD of all subgroups compared to those <65 years old without MetS (reference subgroup). Limitations of this study include the possibilities of misdiagnosis of PD and reverse causality.

**Conclusions:**

Our population-based large-scale cohort study suggests that MetS and its components may be risk factors of PD development.

## Abstract 2

In a cohort study using data from the South Korean National Health Insurance Service, Seon Mee Kim and colleagues investigate associations between components of metabolic syndrome and risk of Parkinson's disease.

## Author summary

**Why was this study done?:**

- Recent evidence has indicated that components of metabolic syndrome (MetS) may contribute to the pathophysiology of Parkinson disease (PD).
- Longitudinal studies regarding the association between MetS and the development of PD are limited.
- Several prospective studies investigating associations between each component of MetS and incident PD have reported inconsistent results.

**What did researchers do and find?:**

- We analyzed the health checkup data of the entire South Korean population aged ≥40 years provided by the Korean National Health Insurance Service between 2009 and 2012.
- Multivariable Cox proportional hazards regression models were used to evaluate the association of MetS and its components with the risk of incident PD, with mean follow-up duration of 5.3 years.
- Our analysis indicated that individuals with MetS had a 24% higher risk of incident PD than individuals without MetS, and each MetS component was positively associated with PD risk.
- Incidence and risk of PD increased gradually with the number of MetS components individuals had.

**What do these findings mean?:**

- MetS and its components might be considered risk factors for PD development.
- Our results show that MetS components are positively associated with increased PD risk and that as the number of components increases, so does the PD risk.
- Optimal control of MetS and its components may reduce the risk of incident PD, and this possibility warrants further investigation.

## Introduction

Metabolic syndrome (MetS) refers to a cluster of several interrelated risk factors for cerebrocardiovascular diseases that result in insulin resistance. These metabolic abnormalities frequently coexist, and MetS is prevalent among patients with obesity and/or a sedentary lifestyle [1,2]. MetS prevalence has been continually increasing in recent decades, globally and in the Republic of Korea (South Korea), due to the obesity epidemic [3]. Each metabolic abnormality predicts both type 2 diabetes and cardiovascular disease, and having a cluster of the abnormalities imposes additional risk in addition to the risks associated with the individual abnormalities [1,2]. In addition, MetS increases all-cause mortality risk and the burden of healthcare costs [4]. Recent evidence has indicated that increased oxidative stress is a major characteristic of MetS-related diseases [5]. Therefore, components of MetS may contribute to the pathophysiology of Parkinson disease (PD), which also shows high levels of reactive oxygen species [6].

PD is a frequent neurodegenerative disease and is considered a leading chronic disease worldwide. PD affects 1 out of 800 individuals worldwide, and PD prevalence is expected to double to over 9 million patients by 2030 due to aging. The increasing prevalence of PD has a substantial impact on morbidity, mortality, and healthcare costs [7]. Prior studies have attempted to uncover the risk factors for incident PD. Recently, the possible role of MetS and its components in PD development has been highlighted. Growing evidence indicates that several mechanistic pathways (such as oxidative stress, lipid pathway alteration, and increased inflammation related to abnormal protein deposition) in neurodegenerative diseases including Alzheimer disease and PD share several elements with the systemic metabolic dysfunction observed in obesity and MetS [8,9]. Furthermore, anti-obesity or metabolically protective therapies have been suggested to be beneficial for patients at risk of neurodegenerative diseases [10]. Thus, we hypothesized that neurodegenerative diseases and metabolic abnormalities are linked due to their shared mechanistic pathophysiology. However, longitudinal studies of the association between MetS and the development of PD are limited. Despite several prospective studies investigating associations between components of MetS and incident PD, results have been inconsistent due to the diverse methodologies implemented. Moreover, only a few studies have exploited nationwide representative cohort data. Our study investigated the association of MetS and its components with PD development using large-scale cohort data from the whole South Korean population.

## Methods

### Data source and study population

Our study was based on the entire South Korean population database provided by the National Health Insurance Service (NHIS), which is a single insurer managed by the Korean government. The NHIS provides a mandatory universal insurance system covering approximately 97% of the South Korean population; the remaining 3% with low income is covered by the Medical Aid Program. Patients subscribed to NHIS pay for 30% of their total medical expenses, and the medical providers must submit claims for reimbursement from the NHIS for the rest. In addition, the NHIS recommends that all insured individuals have a standardized health examination at least every 2 years. Hence, the NHIS retains an extensive health information dataset of approximately 50,000,000 South Koreans regarding demographics, medical treatment, procedures, disease diagnoses according to International Classification of Diseases–10th Revision–Clinical Modification (ICD-10-CM) codes, and health examinations. Since 2015, NHIS has released a dynamic, nationally representative retrospective cohort database consisting of nearly the whole South Korean population, and the database is open to all researchers whose study protocols are approved by the official review committee.

Among individuals ≥40 years old who had undergone a health examination provided by NHIS at least once between January 1, 2009, and December 31, 2012, we excluded those who had a prior diagnosis of PD during the 4 years of washout period before enrollment (*n =* 35,124) and those who had any missing variables (*n =* 674,398). The remaining 17,163,560 eligible individuals (8,215,180 men and 8,948,380 women) were included in the analyses and followed until the date of death or until December 31, 2015. Newly diagnosed PD was identified based on the ICD-10-CM code for PD (G20) and the PD registration code (V124) (the South Korean government has implemented a registration program for copayment reduction of up to 10% for rare intractable diseases including PD since 2006).

This study adhered to the tenets of the Declaration of Helsinki and was approved by the Institutional Review Board of Sahmyook Medical Center (No. SYMC IRB 1706–04). The requirement for written informed consent was waived by the review board because anonymous and de-identified information was used for analysis.

### Assessment and definitions

Detailed information of individuals’ demographics and lifestyle was obtained through standardized self-reporting questionnaires. Income level was dichotomized at the lower 20%. Smoking status was classified as non-smoker, ex-smoker, or current smoker. Individuals who consumed ≥30 g of alcohol per day were defined as heavy alcohol consumers [11]. Physical activity was categorized based on the frequency per week of strenuous exercise performed for at least 20 minutes (none, 1–4 times/week, or ≥5 times/week). Baseline comorbidities (hypertension, diabetes mellitus [DM], dyslipidemia, ischemic heart disease, and stroke) were identified based on the combination of past medical history and ICD-10-CM and prescription codes.

The health examination provided by NHIS includes anthropometric and laboratory measurements. Height, weight, and waist circumference (WC) were measured, and body mass index (BMI) was calculated by dividing weight (kg) by height (m) squared. Systolic and diastolic blood pressure (BP) were measured in a seated position after at least 5 minutes rest. Blood sampling was conducted after overnight fasting, and serum levels of glucose, total cholesterol, triglycerides, high-density lipoprotein cholesterol (HDL-C), low-density lipoprotein cholesterol (LDL-C), and creatinine were measured. MetS was defined based on the modified criteria of the National Cholesterol Education Program Adult Treatment Panel III, while the Asian-specific WC cutoff was adopted for abdominal obesity [2,12]. Individuals with at least 3 of the following components were diagnosed with MetS: (i) WC ≥ 90 cm for men or ≥85 cm for women; (ii) serum triglycerides ≥ 1.70 mmol/l or treatment with lipid-lowering medication; (iii) serum HDL-C < 1.04 mmol/l for men or <1.30 mmol/l for women or treatment with lipid-lowering medication; (iv) systolic BP ≥ 130 mm Hg, diastolic BP ≥ 85 mm Hg, or treatment with antihypertensive medication; and (v) fasting plasma glucose ≥ 5.55 mmol/l or use of hypoglycemic agents. We defined lipid-lowering medication use as at least 1 claim per year for lipid-lowering medication prescription under ICD-10-CM code E78; however, the specific lipid-lowering medication could not be identified. Estimated glomerular filtration rate (eGFR) was calculated using the equation from the Modification of Diet in Renal Disease (MDRD) study: eGFR = 175 × serum creatinine^−1.154^ × age^−0.203^, further multiplied by 0.742 for women [13]. We defined eGFR < 60 ml/min/1.73 m^2^ as chronic kidney disease (CKD) [14].

### Statistical analysis

Statistical analyses were conducted using SAS software (version 9.2; SAS Institute, Cary, NC, US). Baseline characteristics of study participants according to the presence of MetS are presented as mean ± standard deviation for continuous variables and number (percentage) for categorical variables. Values were compared using the independent *t* test for continuous variables and the chi-squared test for categorical variables. Incidence rates of PD were calculated by dividing the number of events by 1,000 person-years. Cox proportional hazards analyses were performed to evaluate the association of MetS and its components with incident PD, and hazard ratios (HRs) and 95% confidence intervals (CIs) were calculated. Model 1 was adjusted for age and sex. Model 2 was additionally adjusted for smoking status, alcohol consumption, physical activity, and income. Model 3 was additionally adjusted for BMI, eGFR, and history of stroke. Given the competing risks of PD and death, as recommended by a reviewer, a competing risk regression model was considered using the Fine and Gray method [15]. Kaplan–Meier curves show the cumulative incidence probability of PD, and a log-rank test was performed to examine the association of the number of MetS components with the risk of PD. We also evaluated the risk of incident PD according to the number of MetS components individuals had using Cox proportional hazards analyses. Stratified analyses according to sex and age were also performed. A *p-*value < 0.05 was considered statistically significant. The text from our study proposal is provided in S1 Text.

## Results

### Baseline characteristics

Study participants were followed up until December 31, 2015, with an average follow-up duration of 5.3 ± 1.2 years. At baseline, 5,848,508 individuals (34.1% of total population) were diagnosed with MetS. Table 1 shows the baseline characteristics of the study population according to the presence of MetS. Mean age was 58.1 ± 11.0 years in the MetS group and 51.9 ± 10.3 in the group without MetS. The proportion of men was higher in the MetS group than the non-MetS group. Individuals with MetS exhibited higher mean values of cardiometabolic parameters such as BMI, WC, BP, fasting plasma glucose, serum total cholesterol, triglycerides, and LDL-C compared to those without MetS. The mean values of HDL-C and eGFR were lower in individuals with MetS than those without. The proportion of non-smokers and of people with regular physical activity was higher in the non-MetS group than the MetS group, and heavy alcohol consumption was higher in the MetS group compared to the non-MetS group. Patients in the MetS group were more likely to have a higher prevalence of hypertension, DM, dyslipidemia, CKD, ischemic heart disease, and stroke. The results of the comparison of baseline characteristics between included individuals and those who were excluded due to missing values are shown in S1 Table. Although the *p-*values calculated were significant except for comorbidities such as dyslipidemia and ischemic heart disease, this appears to result from the very large sample size. There seems to be no actual difference in baseline characteristics between the 2 groups; thus, we do not suspect selection bias.

**Table 1:** Baseline characteristics according to the presence of MetS.

Characteristic	Without MetS	With MetS
***n***	11,315,052	5,848,508
**Age (years)**	51.9 ± 10.3	58.1 ± 11.0
**Sex (male)**	5,413,095 (47.8)	2,802,085 (47.9)
**BMI (kg/m**^**2**^**)**	23.1 ± 2.7	25.6 ± 3.1
**WC (cm)**	78.3 ± 7.9	86.4 ± 8.0
**Systolic BP (mm Hg)**	120.2 ± 14.4	131.2 ± 15.1
**Diastolic BP (mm Hg)**	75.0 ± 9.7	80.7 ± 10.1
**Fasting glucose (mmol/l)**	5.2 ± 1.0	6.2 ± 1.8
**Total cholesterol (mmol/l)**	5.1 ± 0.9	5.3 ± 1.1
**Triglycerides (mmol/l)**	1.1 (0.8–1.5)	1.9 (1.3–2.6)
**HDL-C (mmol/l)**	1.5 ± 0.4	1.3 ± 0.4
**LDL-C (mmol/l)**	3.0 ± 0.8	3.0 ± 1.0
**Creatinine (μmol/l)**	83.1 ± 61.0	85.8 ± 60.1
**eGFR (ml/min/1.73 m**^**2**^**)**	88.3 ± 34.9	83.9 ± 34.0
**Smoking status**		
Non-smoker	7,271,249 (64.3)	3,726,144 (63.7)
Ex-smoker	1,629,522 (14.4)	941,918 (16.1)
Current smoker	2,414,281 (21.3)	1,180,446 (20.2)
**Alcohol consumption**		
Non-drinker	6,526,328 (57.7)	3,623,613 (62.0)
Light to moderate drinker	4,138,579 (36.6)	1,797,711 (30.7)
Heavy drinker	650,145 (5.8)	427,184 (7.3)
**Regular exerciser**^a^	5,666,903 (50.1)	2,674,156 (45.7)
**Low income (lower 20%)**	2,947,306 (26.1)	1,519,123 (26.0)
**Comorbidities**		
Hypertension	2,099,454 (18.6)	3,615,557 (61.8)
Diabetes mellitus	485,845 (4.3)	1,568,771 (26.8)
Dyslipidemia	1,284,958 (11.4)	2,809,269 (48.0)
Chronic kidney disease	540,215 (4.8)	628,989 (10.8)
History of ischemic heart disease	153,268 (2.2)	310,413 (7.2)
History of stroke	92,402 (1.4)	127,363 (3.0)

Values are presented as mean ± standard deviation or number (percentage), except for triglycerides, which are presented as median (interquartile range) using the Wilcoxon rank-sum test.

^a^Strenuous exercise performed for at least 20 minutes ≥1 time/week.

BMI, body mass index; BP, blood pressure; eGFR, estimated glomerular filtration rate; HDL-C, high-density lipoprotein cholesterol; LDL-C, low-density lipoprotein cholesterol; MetS, metabolic syndrome; WC, waist circumference.

### PD risk according to the presence of MetS and its components

A total of 44,205 individuals were diagnosed with PD during the follow-up period, and the incidence rate of PD in the MetS group was approximately 2.2 times higher than that in the non-MetS group. An increased risk of PD development was observed in the MetS group compared with the non-MetS group in all models (HR, 95% CI: model 1, 1.29, 1.27–1.32; model 2, 1.26, 1.24–1.29; model 3, 1.24, 1.21–1.27). Each component of MetS showed a similar association with incidence of PD, even after adjustment for confounding variables. Individuals with abdominal obesity or hypertriglyceridemia had approximately 13% higher risk of PD compared with those without (model 3, HR, 95% CI: 1.13, 1.10–1.16 and 1.13, 1.10–1.15, respectively). Individuals with low HDL-C had a HR of 1.23 (95% CI 1.20–1.25) for PD in model 3. High BP and fasting plasma glucose were also significantly associated with increased risk of PD (model 3, HR, 95% CI: 1.05, 1.03–1.08 and 1.21, 1.18–1.23, respectively) (Table 2). In a competing risk analysis accounting for death as a competing risk, the results were mostly the same as in the main findings in Table 2 (S2 Table).

**Table 2:** HR and 95% CI of incident PD according to MetS and its components.

MetS or component	Events	Person-years	Incidence rate^a^	HR (95% CI)		
				Model 1^b^	Model 2^c^	Model 3^d^
**MetS**						
No	20,697	60,145,059	0.34	1 (ref)	1 (ref)	1 (ref)
Yes	23,508	31,162,667	0.75	1.29 (1.27–1.32)	1.26 (1.24–1.29)	1.24 (1.21–1.27)
**WC (cm)**						
M <90, F <85	29,358	70,421,553	0.42	1 (ref)	1 (ref)	1 (ref)
M ≥90, F ≥85	14,847	20,886,173	0.71	1.21 (1.19–1.23)	1.15 (1.12–1.18)	1.13 (1.10–1.16)
**Serum triglycerides (mmol/l)**						
Low (<1.70)	22,772	55,327,410	0.41	1 (ref)	1 (ref)	1 (ref)
High (≥1.70)	21,433	35,980,316	0.60	1.16 (1.13–1.18)	1.14 (1.12–1.16)	1.13 (1.10–1.15)
**Serum HDL-C (mmol/l)**						
High (M ≥1.04, F ≥1.30)	22,784	60,426,547	0.38	1 (ref)	1 (ref)	1 (ref)
Low (M <1.04, F <1.30)	21,421	30,881,180	0.69	1.27 (1.25–1.30)	1.24 (1.22–1.27)	1.23 (1.20–1.25)
**BP**						
Normal	13,460	45,437,604	0.30	1 (ref)	1 (ref)	1 (ref)
High^e^	30,745	45,870,122	0.67	1.10 (1.08–1.13)	1.07 (1.04–1.09)	1.05 (1.03–1.08)
**Plasma fasting glucose**						
Normal	22,396	57,554,094	0.39	1 (ref)	1 (ref)	1 (ref)
High^f^	21,809	33,753,632	0.65	1.23 (1.21–1.26)	1.21 (1.19–1.24)	1.21 (1.18–1.23)

^a^PD incidence per 1,000 person-years.

^b^Model 1 was adjusted for age and sex.

^c^Model 2 was adjusted for age, sex, smoking status, alcohol consumption, physical activity, and income.

^d^Model 3 was adjusted for age, sex, smoking status, alcohol consumption, physical activity, income, body mass index, estimated glomerular filtration rate, and history of stroke.

^e^Systolic BP ≥ 130 mm Hg, diastolic BP ≥ 85 mm Hg, or treatment with antihypertensive medication.

^f^Plasma fasting glucose ≥ 5.55 mmol/l or use of hypoglycemic agents.

BP, blood pressure; CI, confidence interval; F, females; HDL-C, high-density lipoprotein cholesterol; HR, hazard ratio; M, males; MetS, metabolic syndrome; PD, Parkinson disease; WC, waist circumference.

### Incidence and risk of PD according to the number of MetS components

**Fig 1:**
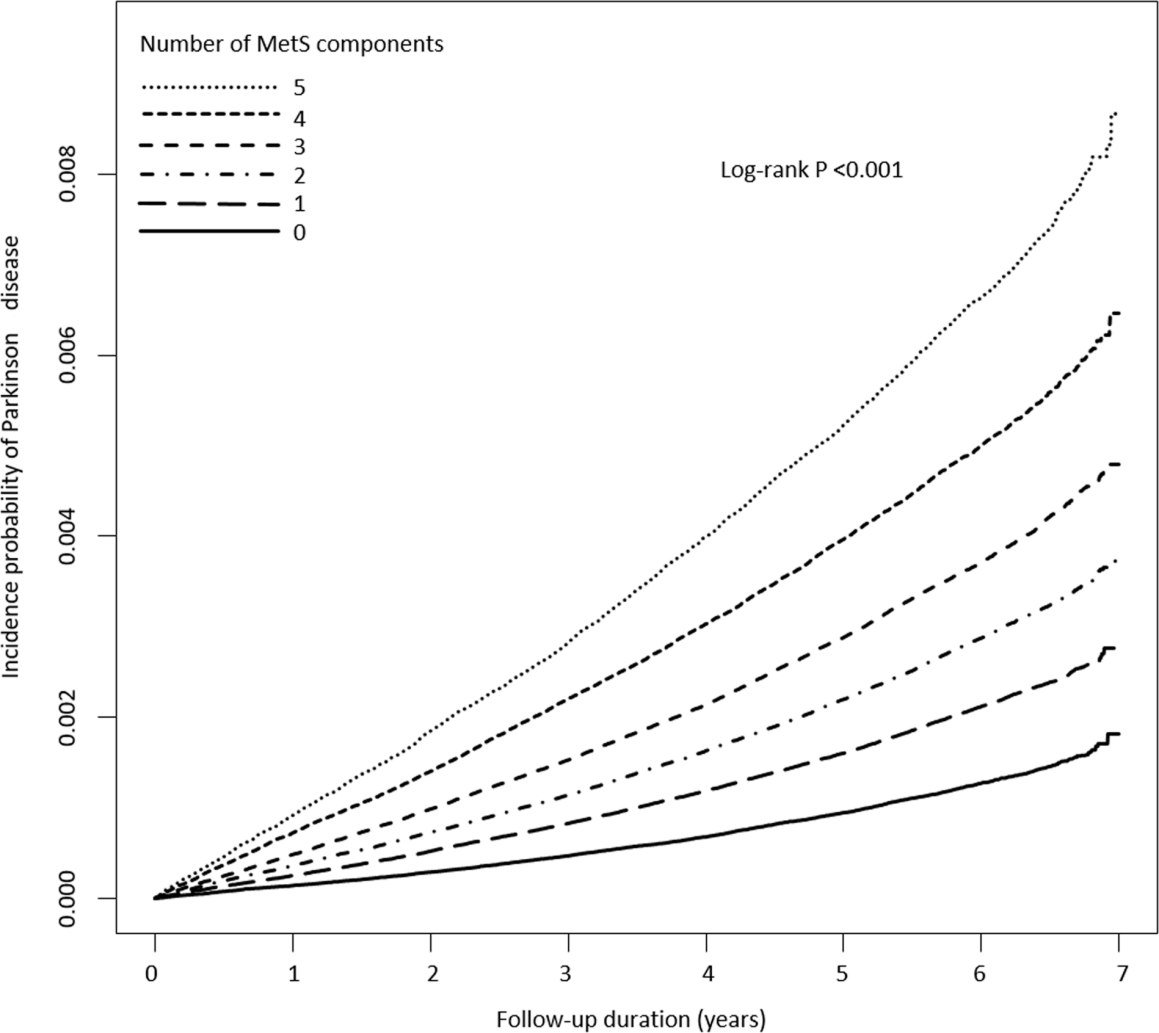
Kaplan–Meier curves of incidence probability of Parkinson disease (PD) for up to 7 years according to the number of metabolic syndrome (MetS) components. Having an increased number of MetS components was associated with increased risk of PD development during the follow-up period compared to having no components (log-rank *p <* 0.001).

Fig 1 and Table 3 show the longitudinal associations between the number of MetS components individuals had and PD incidence. The Kaplan–Meier curve in Fig 1 presents the incidence probability of PD according to the number of MetS components compared to the group without any components. PD incidence was positively correlated with the number of MetS components (log-rank *p <* 0.001). The HR for incident PD compared to people without any MetS components gradually increased with the number of components (*p* for trend < 0.001) (Table 3). These associations persisted even after adjusting for potential confounding variables. Individuals with 3 MetS components were at 31% higher risk of PD, and those with all 5 components were at 66% higher risk, compared to those without any components (model 3).

**Table 3:** HR and 95% CI of incident PD according to the number of MetS components individuals have.

Number of MetS components	Events	Person-years	Incidence rate^a^	HR (95% CI)		
				Model 1^b^	Model 2^c^	Model 3^d^
0	3,522	17,378,573	0.20	1 (ref)	1 (ref)	1 (ref)
1	7,596	22,205,335	0.34	1.10 (1.06–1.15)	1.10 (1.06–1.15)	1.13 (1.07–1.19)
2	9,579	20,561,151	0.47	1.18 (1.14–1.23)	1.18 (1.14–1.23)	1.20 (1.14–1.26)
3	9,696	15,925,211	0.61	1.31 (1.26–1.36)	1.31 (1.26–1.36)	1.31 (1.25–1.38)
4	8,735	10,589,923	0.82	1.50 (1.44–1.56)	1.49 (1.43–1.56)	1.49 (1.42–1.57)
5	5,077	4,647,533	1.09	1.70 (1.63–1.78)	1.69 (1.61–1.77)	1.66 (1.57–1.76)
*p* for trend				<0.001	<0.001	<0.001

^a^PD incidence per 1,000 person-years.

^b^Model 1 was adjusted for age and sex.

^c^Model 2 was adjusted for age, sex, smoking status, alcohol consumption, physical activity, and income.

^d^Model 3 was adjusted for age, sex, smoking status, alcohol consumption, physical activity, income, body mass index, estimated glomerular filtration rate, and history of stroke.

CI, confidence interval; HR, hazard ratio; MetS, metabolic syndrome; PD, Parkinson disease.

### Risk of incident PD according to the combination of age and MetS status

**Fig 2:**
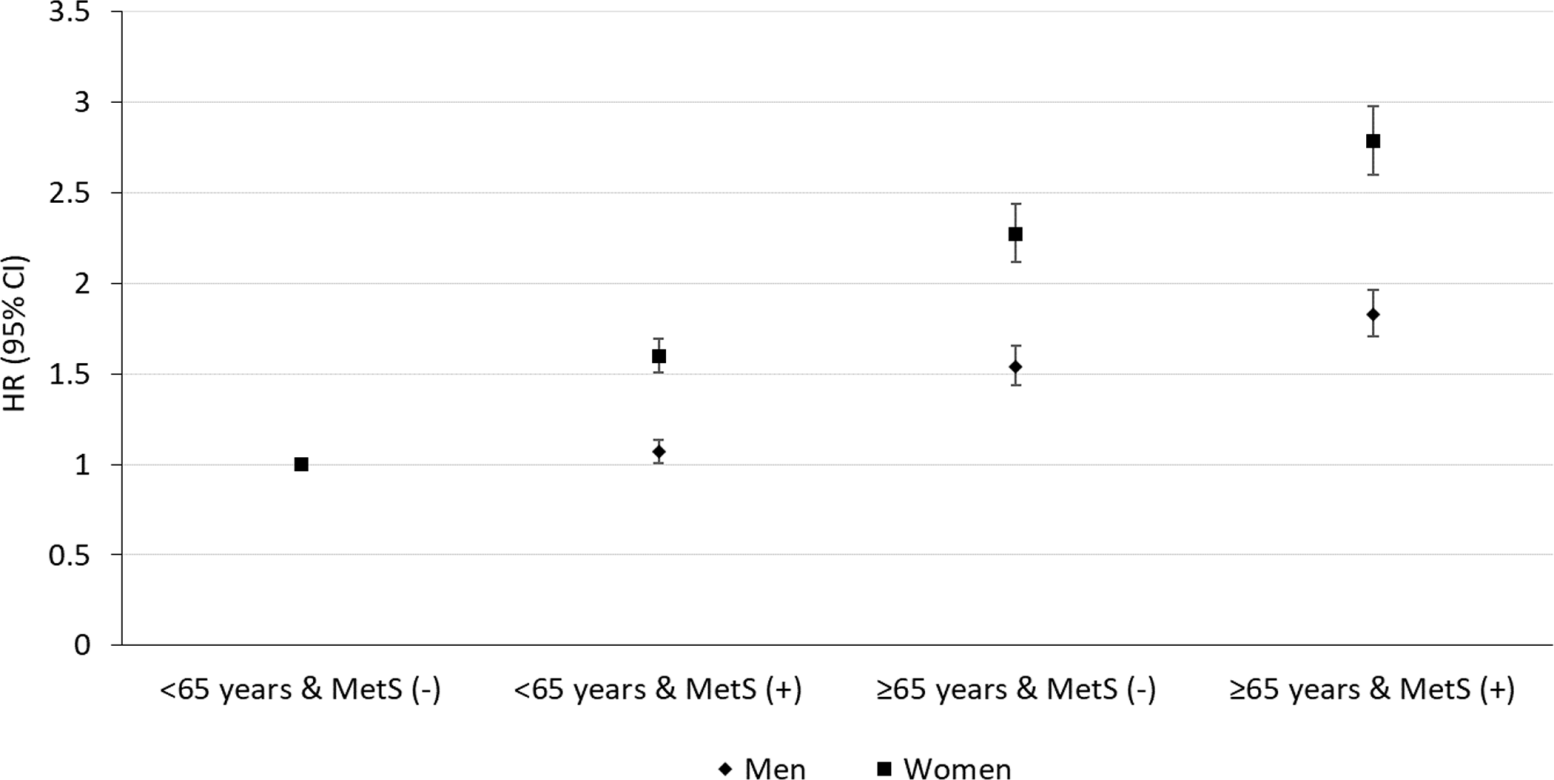
Combined effects of age and metabolic syndrome (MetS) status on the risk of incident Parkinson disease (PD). Increased hazard ratios (HRs) were observed in individuals <65 years old with MetS, individuals ≥65 years old without MetS, and individuals ≥65 years old with MetS compared to those <65 years old without MetS (*p <* 0.001). Interaction between age and MetS on the risk of PD was significant (*p* for interaction < 0.001).

Fig 2 shows the combined effects of age and MetS on the incident PD risk after all potential confounding variables were adjusted for. There was a significant interaction between age and MetS on the risk of PD (*p* for interaction < 0.001). Compared to people aged <65 years without MetS, we observed a gradual increase in the HR of PD for individuals <65 years with MetS, those ≥65 years without MetS, and those ≥65 years with MetS, in both sexes (*p <* 0.001). People with both old age (≥65 years) and MetS had the highest HR of incident PD (HR, 95% CI: 1.83, 1.71–1.96 in men and 2.78, 2.60–2.98 in women).

## Discussion

Our population-based large-scale cohort study revealed that the incidence rate of PD was approximately 2.2 times greater for people with MetS compared to those without MetS over a 5.3-year follow-up period and that individuals with MetS had a 24% higher risk of incident PD. The presence of each MetS component was also associated with increased risk of PD development, and individuals with a higher number of MetS components were at higher risk of incident PD. These associations persisted even after adjusting for potential confounding variables. Furthermore, increased risk of PD was observed for individuals ≥65 years old compared to younger individuals (<65 years); older individuals with MetS showed the greatest risk of PD. These associations were particularly prominent in women.

Our findings suggest that MetS may be a risk factor for incident PD. Our results also show that even individual components of MetS are positively associated with increased PD risk and that as the number of components increases, so does PD risk. Therefore, our study shows important clinical implications for MetS and its components in PD development.

There is limited previous evidence regarding the impact of MetS on the risk of PD development. To the best of our knowledge, only 1 case-control study and 1 prospective study have been performed, showing mixed results. The case-control study compared 80 PD patients (with BMI ≥ 18.5 kg/m^2^) to 80 controls and reported that there was a lack of association between MetS and PD [16]. However, apart from the limitations deriving from the study design and the small sample size, this study was limited by an inclusion bias: the PD group included did not represent the whole patient population, and the controls were admitted to a medical center due to their excessive body weight, a condition that may be associated with other comorbidities. The prospective study of 6,641 adults based on the Mini-Finland Health Survey with a follow-up duration of 30 years reported that the adjusted relative risk of PD was 0.5 (95% CI 0.30–0.83) for individuals with MetS compared to those without [17]. This study suggested that elevated levels of serum triglycerides and fasting plasma glucose were related to low PD incidence and that there was an inverse association between MetS and PD mainly attributable to serum triglyceride concentration. Although the findings from this study are at odds with ours, the methodology followed was different from ours, with a smaller sample size and a longer follow-up duration, which likely caused unrelated changes to the patients’ health status. Our study, based on a large nationwide population cohort with a relatively short follow-up period, provides novel insight into the association between MetS and PD.

Several previous epidemiological studies have evaluated individual cardiovascular risk factors such as hypertension, DM, dyslipidemia, and obesity—which are similar to the components of MetS studied here—as risk factors for PD. These studies have produced conflicting findings, and, moreover, the underlying mechanisms that may explain the observed associations of MetS and its components with incident PD risk are currently unclear. However, a possible mechanism may be deduced from evidence regarding the association between obesity and PD risk. A 30-year follow-up study of men in the Honolulu Heart Program found that high triceps skinfold thickness in midlife is associated with future PD risk [18]. People with obesity have lower dopamine receptor availability than non-obese people; this may cause compensatory increases in turnover of dopamine, and lead to increased oxidative stress and neuronal death [18,19]. Conversely, a recent study using 2-sample Mendelian randomization reported that genetic variants known to influence BMI appear to be associated with lower risk of PD [20]. However, our findings revealed that abdominal obesity is positively associated with incident PD risk even after adjusting for BMI. The low-grade chronic and systemic inflammation prevalently observed in abdominal obesity and MetS is possibly associated with increased PD risk [21]. Additionally, animal studies proved that a high-fat diet is accompanied by dopamine-specific toxin exposure and may reduce the threshold for developing PD [22–24].

No previous cohort studies have investigated the effect of blood triglycerides and HDL-C on PD risk, except for the aforementioned Finnish prospective study [17]. A Swedish longitudinal nested case-control study reported that high blood triglyceride levels were less frequent in PD patients than controls; however, this association was attenuated after adjustment for smoking [25]. A prospective study found that high serum levels of total cholesterol and triglycerides (in men) were associated with an elevated risk of developing restless legs syndrome, which may constitute a possible preclinical marker of PD [26,27]. Additionally, the protective effects of statins on PD risk may partly explain the association of high serum triglyceride and low serum HDL-C levels with increased PD risk [28]. Meanwhile, several studies on serum total cholesterol have reported conflicting findings, and these inconsistent findings may be explained by the various time points at which cholesterol levels were measured and personality changes during the premotor phase of PD [29,30]. However, high serum triglyceride and low serum HDL-C levels are closely related to insulin resistance, which appears to occur analogously in the brains of PD patients because a defect in the insulin signaling pathway may contribute to the pathogenesis of PD [31].

The association between hypertension and PD is still unclear. In various studies, hypertension was less frequent in PD patients [32], hypertension showed no difference compared with healthy people [33], or history of hypertension was found not to be associated with PD risk [29]. However, a Finnish retrospective cohort study reported that high-normal BP and hypertension were associated with an increased PD risk in women [34]. Although the mechanisms linking hypertension and elevated BP to PD are still unclear, persistent hypertension can cause ischemic cerebrovascular lesions. Cerebral ischemia possibly activates the dopaminergic pathway due to decreased expression of nicotinic acetylcholine receptors, and plays a role in the clinical expression and deterioration of idiopathic PD symptomatology [35,36]. Long-term elevated BP likely causes hypertensive vasculopathy in brain structures, which may influence the dopaminergic cells and break the links between neurons in the substantia nigra and the putamen (striatum) [37].

Our results are supported by the fact that DM may be a risk factor for PD. Most epidemiological evidence supports the positive association between DM and PD risk, although there are discrepancies that may be explained by residual confounders [31]. A prospective cohort study suggested that type 2 DM is independently associated with PD risk after adjusting for various confounding variables [38]. Furthermore, several studies have suggested that even insulin resistance or prediabetes negatively affects the course of PD [39–41]. Pathophysiological mechanisms regarding the link between hyperglycemia and incident PD are speculative other than that they share cellular mechanisms such as mitochondrial dysfunction and decreased expression of the transcriptional regulator PPARγ coactivator 1α (PGC1α), which stimulates mitochondrial biogenesis and respiration [42,43].

Interestingly, our study revealed a combined effect of age and MetS on PD development. Aging is the most important risk factor for PD, and our study found that people with older age and MetS had the highest risk for incident PD. Even older people without MetS showed higher risk of PD development than younger individuals with MetS. In addition, the HRs were higher among women than men in all subgroups categorized according to age and MetS status. This is in contrast to previous studies that showed that males were more susceptible to PD, possibly due to the interplay between sex-specific hormones and genes [44]. However, increased oxidative stress, which is the common mechanism leading to MetS and PD, especially in postmenopausal women, may explain the reported associations [45].

In this way, several epidemiological studies and additional animal and experimental studies are consistent with our findings. However, discordances between previous studies and the current study are recognized, suggesting that residual confounding or modifying factors may modulate the association of MetS and its components with PD risk. Thus, further epidemiological, basic science, and clinical research is still needed on the relationship between MetS and PD. The current study, in which each MetS trait appears to be associated with increased incident PD risk, supports the data on the link between MetS and PD, implicating that MetS and its components may contribute to the pathophysiology of PD and act as risk factors for PD. The risk of PD may be exacerbated by metabolism-related dysfunction related to MetS, and any interventions to control MetS traits in the general population could be beneficial not only for common chronic conditions related to MetS, but also for PD.

Several limitations should be mentioned regarding the interpretation of our results. First, because the NHIS database relies on physicians’ assignment of a diagnostic code for PD, there may be a possibility of misdiagnosis of PD, which could result in under- or overestimation. Also, individuals with non-motor symptoms who were yet to be diagnosed with PD at baseline could have been more likely to participate in the NHIS health examinations, resulting in selection bias. Second, because this study was not prospectively designed, causality cannot be determined. Individuals with prior diagnosis of PD during the 4 years before enrollment were excluded to minimize the possibility of reverse causality. However, there is still a possibility of reverse causality based on the long prodromal phase of PD. Third, due to lack of data, we did not consider dietary factors that may be potentially related to MetS and its components. Fourth, the duration of MetS, which possibly influences PD risk, could not be considered. Fifth, we could not distinguish the specific lipid-lowering medications used in the diagnosis of the MetS components hypertriglyceridemia and low HDL-C. Finally, our findings from the Korean population cannot be extrapolated to other ethnicities.

Nevertheless, our study has a major strong point, because it is a very large-scale cohort study that evaluated the influence of MetS and its components on PD. Our study provides the first evidence to our knowledge that MetS and its components constitute risk factors for PD in the general population, because the NHIS database includes the entire South Korean population. Further, we had comprehensive ascertainment of coexisting illnesses, allowing for adjustment for potential confounders.

In conclusion, we found that MetS and its components are independent risk factors for PD development. Careful monitoring of neurological symptoms related to PD seems to be helpful for patients with MetS, and assessment of MetS may be considered when encountering a newly diagnosed parkinsonism. Future studies are warranted to examine whether control of MetS and its components can decrease the risk of PD development.

## Supporting information

S1 RECORD Checklist RECORD checklist of items that should be included in reports of cohort studies. (DOCX)

S1 Text Study protocol when applying for use of the data (November 15, 2017). (DOCX)

S1 Table Comparison of baseline characteristics between included individuals and individuals excluded due to missing values. (DOCX)

S2 Table A competing risk analysis model accounting for death as a competing risk. (DOCX)

## Abbreviations

BMI: body mass index
BP: blood pressure
CI: confidence interval
CKD: chronic kidney disease
DM: diabetes mellitus
eGFR: estimated glomerular filtration rate
HDL-C: high-density lipoprotein cholesterol
HR: hazard ratio
LDL-C: low-density lipoprotein cholesterol
MetS: metabolic syndrome
NHIS: National Health Insurance Service
PD: Parkinson disease
WC: waist circumference

## References

[1] Simmons RK, Alberti KG, Gale EA, Colagiuri S, Tuomilehto J, Qiao Q, et al The metabolic syndrome: useful concept or clinical tool? Report of a WHO Expert Consultation. Diabetologia. 2010;53:600–5. 10.1007/s00125-009-1620-4 20012011

[2] Alberti KG, Eckel RH, Grundy SM, Zimmet PZ, Cleeman JI, Donato KA, et al Harmonizing the metabolic syndrome: a joint interim statement of the International Diabetes Federation Task Force on Epidemiology and Prevention; National Heart, Lung, and Blood Institute; American Heart Association; World Heart Federation; International Atherosclerosis Society; and International Association for the Study of Obesity. Circulation. 2009;120:1640–5. 10.1161/CIRCULATIONAHA.109.192644 19805654

[3] Lim S, Shin H, Song JH, Kwak SH, Kang SM, Won Yoon J, et al Increasing prevalence of metabolic syndrome in Korea: the Korean National Health and Nutrition Examination Survey for 1998–2007. Diabetes Care. 2011;34:1323–8. 10.2337/dc10-2109 21505206 PMC3114326

[4] Malik S, Wong ND, Franklin SS, Kamath TV, L’Italien GJ, Pio JR, et al Impact of the metabolic syndrome on mortality from coronary heart disease, cardiovascular disease, and all causes in United States adults. Circulation. 2004;110:1245–50. 10.1161/01.CIR.0000140677.20606.0E 15326067

[5] Bhatti JS, Bhatti GK, Reddy PH. Mitochondrial dysfunction and oxidative stress in metabolic disorders—a step towards mitochondria based therapeutic strategies. Biochim Biophys Acta. 2017;1863:1066–77. 10.1016/j.bbadis.2016.11.010 27836629 PMC5423868

[6] Zhang P, Tian B. Metabolic syndrome: an important risk factor for Parkinson’s disease. Oxid Med Cell Longev. 2014;2014:729194 10.1155/2014/729194 24955210 PMC4052080

[7] Ascherio A, Schwarzschild MA. The epidemiology of Parkinson’s disease: risk factors and prevention. Lancet Neurol. 2016;15:1257–72. 10.1016/S1474-4422(16)30230-7 27751556

[8] Perl DP, Olanow CW, Calne D. Alzheimer’s disease and Parkinson’s disease: distinct entities or extremes of a spectrum of neurodegeneration? Ann Neurol. 1998;44(3 Suppl 1):S19–31. 9749570 10.1002/ana.410440705

[9] Mattson MP, Pedersen WA, Duan W, Culmsee C, Camandola S. Cellular and molecular mechanisms underlying perturbed energy metabolism and neuronal degeneration in Alzheimer’s and Parkinson’s diseases. Ann N Y Acad Sci. 1999;893:154–75. 10672236 10.1111/j.1749-6632.1999.tb07824.x

[10] Ashrafian H, Harling L, Darzi A, Athanasiou T. Neurodegenerative disease and obesity: what is the role of weight loss and bariatric interventions? Metab Brain Dis. 2013;28(3):341–53. 10.1007/s11011-013-9412-4 23653255

[11] Agarwal DP. Cardioprotective effects of light-moderate consumption of alcohol: a review of putative mechanisms. Alcohol Alcohol. 2002;37:409–15. 12217928 10.1093/alcalc/37.5.409

[12] Kim MK, Lee WY, Kang JH, Kang JH, Kim BT, Kim SM, et al 2014 clinical practice guidelines for overweight and obesity in Korea. Endocrinol Metab (Seoul). 2014;29:405–9. 25559568 10.3803/EnM.2014.29.4.405 PMC4285036

[13] Levey AS, Coresh J, Greene T, Stevens LA, Zhang YL, Hendriksen S, et al Using standardized serum creatinine values in the modification of diet in renal disease study equation for estimating glomerular filtration rate. Ann Intern Med. 2006;145:247–54. 16908915 10.7326/0003-4819-145-4-200608150-00004

[14] Levey AS, Coresh J, Balk E, Kausz AT, Levin A, Steffes MW, et al National Kidney Foundation practice guidelines for chronic kidney disease: evaluation, classification, and stratification. Ann Intern Med. 2003;139:137–47. 12859163 10.7326/0003-4819-139-2-200307150-00013

[15] Austin PC, Lee DS, Fine JP. Introduction to the analysis of survival data in the presence of competing risks. Circulation. 2016;133:601–9. 10.1161/CIRCULATIONAHA.115.017719 26858290 PMC4741409

[16] Cereda E, Cassani E, Barichella M, Spadafranca A, Caccialanza R, Bertoli S, et al Low cardiometabolic risk in Parkinson’s disease is independent of nutritional status, body composition and fat distribution. Clin Nutr. 2012;31:699–704. 10.1016/j.clnu.2012.02.004 22402420

[17] Saaksjarvi K, Knekt P, Mannisto S, Lyytinen J, Heliovaara M. Prospective study on the components of metabolic syndrome and the incidence of Parkinson’s disease. Parkinsonism Relat Disord. 2015;21:1148–55. 10.1016/j.parkreldis.2015.07.017 26228080

[18] Abbott RD, Ross GW, White LR, Nelson JS, Masaki KH, Tanner CM, et al Midlife adiposity and the future risk of Parkinson’s disease. Neurology. 2002;59:1051–7. 12370461 10.1212/wnl.59.7.1051

[19] Wang GJ, Volkow ND, Fowler JS. The role of dopamine in motivation for food in humans: implications for obesity. Expert Opin Ther Targets. 2002;6:601–9. 10.1517/14728222.6.5.601 12387683

[20] Noyce AJ, Kia DA, Hemani G, Nicolas A, Price TR, Pablo-Fernandez E, et al Estimating the causal influence of body mass index on risk of Parkinson disease: a Mendelian randomisation study. PLoS Med. 2017;14:e1002314 10.1371/journal.pmed.1002314 28609445 PMC5469450

[21] Bastard JP, Maachi M, Lagathu C, Kim MJ, Caron M, Vidal H, et al Recent advances in the relationship between obesity, inflammation, and insulin resistance. Eur Cytokine Netw. 2006;17:4–12. 16613757

[22] Morris JK, Bomhoff GL, Stanford JA, Geiger PC. Neurodegeneration in an animal model of Parkinson’s disease is exacerbated by a high-fat diet. Am J Physiol Regul Integr Comp Physiol. 2010;299:R1082–90. 10.1152/ajpregu.00449.2010 20702796 PMC2957375

[23] Cano P, Cardinali DP, Rios-Lugo MJ, Fernandez-Mateos MP, Reyes Toso CF, Esquifino AI. Effect of a high-fat diet on 24-hour pattern of circulating adipocytokines in rats. Obesity (Silver Spring). 2009;17:1866–71. 19543212 10.1038/oby.2009.200

[24] Uranga RM, Bruce-Keller AJ, Morrison CD, Fernandez-Kim SO, Ebenezer PJ, Zhang L, et al Intersection between metabolic dysfunction, high fat diet consumption, and brain aging. J Neurochem. 2010;114:344–61. 10.1111/j.1471-4159.2010.06803.x 20477933 PMC2910139

[25] Vikdahl M, Backman L, Johansson I, Forsgren L, Haglin L. Cardiovascular risk factors and the risk of Parkinson’s disease. Eur J Clin Nutr. 2015;69:729–33. 10.1038/ejcn.2014.259 25514902

[26] De Vito K, Li Y, Batool-Anwar S, Ning Y, Han J, Gao X. Prospective study of obesity, hypertension, high cholesterol, and risk of restless legs syndrome. Mov Disord. 2014;29:1044–52. 10.1002/mds.25860 24753235 PMC4501395

[27] Gao X, Schwarzschild MA, O’Reilly EJ, Wang H, Ascherio A. Restless legs syndrome and Parkinson’s disease in men. Mov Disord. 2010;25:2654–7. 10.1002/mds.23256 20737545 PMC3114885

[28] Bai S, Song Y, Huang X, Peng L, Jia J, Liu Y, et al Statin use and the risk of Parkinson’s disease: an updated meta-analysis. PLoS ONE. 2016;11:e0152564 10.1371/journal.pone.0152564 27019096 PMC4809483

[29] Simon KC, Chen H, Schwarzschild M, Ascherio A. Hypertension, hypercholesterolemia, diabetes, and risk of Parkinson disease. Neurology. 2007;69:1688–95. 10.1212/01.wnl.0000271883.45010.8a 17761552 PMC2391077

[30] de Lau LM, Koudstaal PJ, Hofman A, Breteler MM. Serum cholesterol levels and the risk of Parkinson’s disease. Am J Epidemiol. 2006;164:998–1002. 10.1093/aje/kwj283 16905642

[31] Athauda D, Foltynie T. Insulin resistance and Parkinson’s disease: a new target for disease modification? Prog Neurobiol. 2016;145–146:98–120. 10.1016/j.pneurobio.2016.10.001 27713036

[32] Scigliano G, Musicco M, Soliveri P, Piccolo I, Ronchetti G, Girotti F. Reduced risk factors for vascular disorders in Parkinson disease patients: a case-control study. Stroke. 2006;37:1184–8. 10.1161/01.STR.0000217384.03237.9c 16574924

[33] Morano A, Jimenez-Jimenez FJ, Molina JA, Antolin MA. Risk-factors for Parkinson’s disease: case-control study in the province of Caceres, Spain. Acta Neurol Scand. 1994;89:164–70. 8030397 10.1111/j.1600-0404.1994.tb01655.x

[34] Qiu C, Hu G, Kivipelto M, Laatikainen T, Antikainen R, Fratiglioni L, et al Association of blood pressure and hypertension with the risk of Parkinson disease: the National FINRISK Study. Hypertension. 2011;57:1094–100. 10.1161/HYPERTENSIONAHA.111.171249 21536985

[35] Papapetropoulos S, Ellul J, Argyriou AA, Talelli P, Chroni E, Papapetropoulos T. The effect of vascular disease on late onset Parkinson’s disease. Eur J Neurol. 2004;11:231–5. 10.1046/j.1468-1331.2003.00748.x 15061824

[36] Tohgi H, Utsugisawa K, Yoshimura M, Nagane Y, Mihara M. Alterations with aging and ischemia in nicotinic acetylcholine receptor subunits alpha4 and beta2 messenger RNA expression in postmortem human putamen. Implications for susceptibility to parkinsonism. Brain Res. 1998;791:186–90. 9593888 10.1016/s0006-8993(98)00093-6

[37] Greenberg SM, Vernooij MW, Cordonnier C, Viswanathan A, Al-Shahi Salman R, Warach S, et al Cerebral microbleeds: a guide to detection and interpretation. Lancet Neurol. 2009;8:165–74. 10.1016/S1474-4422(09)70013-4 19161908 PMC3414436

[38] Hu G, Jousilahti P, Bidel S, Antikainen R, Tuomilehto J. Type 2 diabetes and the risk of Parkinson’s disease. Diabetes Care. 2007;30:842–7. 10.2337/dc06-2011 17251276

[39] Kotagal V, Albin RL, Muller ML, Koeppe RA, Frey KA, Bohnen NI. Diabetes is associated with postural instability and gait difficulty in Parkinson disease. Parkinsonism Relat Disord. 2013;19:522–6. 10.1016/j.parkreldis.2013.01.016 23462483 PMC3607954

[40] Cereda E, Barichella M, Cassani E, Caccialanza R, Pezzoli G. Clinical features of Parkinson disease when onset of diabetes came first: a case-control study. Neurology. 2012;78:1507–11. 10.1212/WNL.0b013e3182553cc9 22539572

[41] Bosco D, Plastino M, Cristiano D, Colica C, Ermio C, De Bartolo M, et al Dementia is associated with insulin resistance in patients with Parkinson’s disease. J Neurol Sci. 2012;315:39–43. 10.1016/j.jns.2011.12.008 22265943

[42] Aviles-Olmos I, Limousin P, Lees A, Foltynie T. Parkinson’s disease, insulin resistance and novel agents of neuroprotection. Brain. 2013;136:374–84. 10.1093/brain/aws009 22344583

[43] Santiago JA, Potashkin JA. Shared dysregulated pathways lead to Parkinson’s disease and diabetes. Trends Mol Med. 2013;19:176–86. 10.1016/j.molmed.2013.01.002 23375873

[44] Loke H, Harley V, Lee J. Biological factors underlying sex differences in neurological disorders. Int J Biochem Cell Biol. 2015;65:139–50. 10.1016/j.biocel.2015.05.024 26028290

[45] Rodriguez-Perez AI, Valenzuela R, Villar-Cheda B, Guerra MJ, Lanciego JL, Labandeira-Garcia JL. Estrogen and angiotensin interaction in the substantia nigra. Relevance to postmenopausal Parkinson’s disease. Exp Neurol. 2010;224:517–26. 10.1016/j.expneurol.2010.05.015 20580712

