# Supplementary material for: Metabolic syndrome and risk of Parkinson disease: A nationwide cohort study
Source: PLoS Med. 2018 Aug 21;15(8):e1002640. doi: 10.1371/journal.pmed.1002640 (PMC6103502; doi:10.1371/journal.pmed.1002640)
Supplement: S1 Text — (DOCX) [file pmed.1002640.s002.docx]

**S1 Text. Study protocol when applying for use of the data**

**Study contents**

Parkinson's disease is one of the neurodegenerative diseases, which are more heavily dependent on public health in high-income countries with older people and higher socioeconomic levels. Although epidemiological indicators of Parkinson's disease are needed first to establish management measures and identify causes, there is still a lack of nationwide research data. Therefore, through big data analysis provided by the National Health Insurance Corporation, we aims to investigate the prevalence and risk factors of Parkinson's disease across the country. Metabolic syndrome is a common problem, but studies looking at relationships between metabolic syndrome and the risk of Parkinson's are rare and inconsistent. This study aims to explore the associations between metabolic syndrome and the risk of Parkinson's in Korean adults.

**Study methods**

- Inclusion criteria: Extraction of patients with the corresponding ICD-10-CM codes and registration codes
- Exclude Criteria: 1) Failure to meet age standards or 2) has already been diagnosed prior to the observation period
- Analysis Method: Using the Cox proportional risk model to investigate and compare the risk of developing Parkinson's disease by metabolic syndrome and its components

- Subgroup analysis is carried out according to sex and age.

**Expectation effectiveness**- Big Data analysis enable a systematic understanding of modifiable risk factors to prevent Parkinson's disease.

- Can be used as a basis for drawing social attention and further research and presenting various policies to prevent and treat Parkinson's disease
