# Supplementary material for: Metabolic syndrome and risk of Parkinson disease: A nationwide cohort study
Source: PLoS Med. 2018 Aug 21;15(8):e1002640. doi: 10.1371/journal.pmed.1002640 (PMC6103502; doi:10.1371/journal.pmed.1002640)
Supplement: S1 Table — (DOCX) [file pmed.1002640.s003.docx]

**S1 Table. Comparison of baseline characteristics between included individuals and excluded individuals due to missing values**

|  | Included individuals | Excluded individuals due to missing values | P |
| --- | --- | --- | --- |
| N | 17,163,560 | 674,398 |  |
| Age (years) | 53.9 ± 10.9 | 55.3 ± 11.3 | <0.001 |
| Sex (male) | 8,215,180 (47.9) | 30,203 (45.7) | <0.001 |
| BMI (kg/m^2^) | 24.0 ± 3.1 | 24.0 ± 3.0 | <0.001 |
| WC (cm) | 81.1 ± 8.8 | 81.3 ± 8.1 | <0.001 |
| Systolic BP (mmHg) | 123.9 ± 15.5 | 124.7 ± 15.4 | <0.001 |
| Diastolic BP (mmHg) | 77.0 ± 10.2 | 76.9 ± 10.2 | <0.001 |
| Fasting glucose (mg/dL) | 100.1 ± 25.1 | 102.8 ± 27.9 | <0.001 |
| Total cholesterol (mg/dL) | 198.7 ± 37.2 | 197.2 ± 37.1 | <0.001 |
| Triglycerides (mg/dL)^a^ | 117.6 (39.6-349.6) | 121.5 (121-122) | <0.001 |
| HDL-C (mg/dL) | 54.8 ± 16.9 | 54.4 ± 17.0 | <0.001 |
| LDL-C (mg/dL) | 117.0 ± 34.3 | 122.9 ± 53.5 | <0.001 |
| Creatinine (mg/dL) | 0.95 ± 0.69 | 1.10 ± 1.10 | <0.001 |
| eGFR (mL/min/1.73 m^2^) | 86.9 ± 34.7 | 84.9 ± 51.2 | <0.001 |
| Smoking status |  |  | <0.001 |
| Non-smoker | 10,997,393 (64.1) | 31,297 (60.7) |  |
| Ex-smoker | 2,571,440 (15.0) | 8270 (16.0) |  |
| Current smoker | 3,594,727 (20.9) | 12,001 (23.3) |  |
| Alcohol consumption |  |  | <0.001 |
| Non-drinker | 10,149,941 (59.1) | 11,668 (56.9) |  |
| Light to moderate drinker | 5,936,290 (34.6) | 7145 (34.8) |  |
| Heavy drinker | 1,077,329 (6.3) | 1697 (8.3) |  |
| Regular exerciser | 8,341,059 (48.6) | 24,223 (52.8) | <0.001 |
| Income (lower 20%) | 4,466,429 (26.0) | 16,375 (24.8) | <0.001 |
| Comorbidities |  |  |  |
| Hypertension | 5,715,011 (33.3) | 23,655 (35.8) | <0.001 |
| Diabetes mellitus | 2,054,616 (12.0) | 9363 (14.2) | <0.001 |
| Dyslipidemia | 4,094,227 (23.9) | 15,849 (24.0) | 0.370 |
| Chronic kidney disease | 1,169,204 (6.8) | 6316 (9.6) | <0.001 |
| History of ischemic heart disease | 463,681 (2.7) | 931 (1.8) | 0.120 |
| History of stroke | 219,765 (1.3) | 1842 (3.6) | <0.001 |
