# Supplementary material for: Metabolic syndrome and risk of Parkinson disease: A nationwide cohort study
Source: PLoS Med. 2018 Aug 21;15(8):e1002640. doi: 10.1371/journal.pmed.1002640 (PMC6103502; doi:10.1371/journal.pmed.1002640)
Supplement: S2 Table — (DOCX) [file pmed.1002640.s004.docx]

**S2 Table. A competing risk analysis model accounting for death as a competing risk**

|  | HR (95% CI) |  |  |
| --- | --- | --- | --- |
|  | Model 1 | Model 2 | Model 3 |
| MetS |  |  |  |
| No | 1 (ref) | 1 (ref) | 1 (ref) |
| Yes | 1.21 (1.20–1.21) | 1.30 (1.29–1.31) | 1.26 (1.25–1.27) |
| WC (cm) |  |  |  |
| M <90, F <85 | 1 (ref) | 1 (ref) | 1 (ref) |
| M ≥90, F ≥85 | 1.033 (1.026–1.04) | 1.166 (1.156–1.175) | 1.151 (1.14–1.162) |
| Serum triglycerides (mg/dL) |  |  |  |
| Low (<150) | 1 (ref) | 1 (ref) | 1 (ref) |
| High (≥150) | 1.154 (1.147–1.162) | 1.187 (1.179–1.194) | 1.147 (1.139–1.156) |
| Serum HDL-C (mg/dL) |  |  |  |
| High (M ≥40, F ≥50) | 1 (ref) | 1 (ref) | 1 (ref) |
| Low (M <40, F <50) | 1.162 (1.154–1.169) | 1.179 (1.171–1.187) | 1.141 (1.133–1.15) |
| BP |  |  |  |
| Normal | 1 (ref) | 1 (ref) | 1 (ref) |
| High | 1.157 (1.148–1.165) | 1.204 (1.195–1.213) | 1.169 (1.158–1.18) |
| Plasma fasting glucose (mg/dL) |  |  |  |
| Normal | 1 (ref) | 1 (ref) | 1 (ref) |
| High | 1.232 (1.224–1.24) | 1.262 (1.254–1.27) | 1.257 (1.248–1.266) |

Model 1 was adjusted for age and sex.

Model 2 was adjusted for age, sex, smoking status, alcohol consumption, physical activity, and income.

Model 3 was adjusted for age, sex, smoking status, alcohol consumption, physical activity, income, body mass index, estimated glomerular filtration rate, and history of stroke.
